# Editorial: The Unusual Presentation of Thyroid Disorders

**DOI:** 10.3389/fendo.2019.00560

**Published:** 2019-08-20

**Authors:** Giampaolo Papi, Alfredo Pontecorvi

**Affiliations:** ^1^Endocrinology Unit-Northern Area, Azienda USL Modena, Modena, Italy; ^2^Department of Endocrinology, Catholic University of the Sacred Heart, Rome, Italy; ^3^Endocrinology Unit, Fondazione Policlinico Universitario A. Gemelli – IRCCS, Rome, Italy

**Keywords:** nodular thyroid disease, hypothyroidism, hyperthyroidism, thyroid neoplasm, intrathyroidal parathyroid carcinoma, atypical thyroid lesions

Clinical endocrinologists deal every day with patients affected by thyroid diseases and are aware of their multifaceted signs and symptoms. Thus, the “scholar” description of thyroid dysfunction syndrome referring to heart, brain, skin and eyes as the main targeted sites, sometimes does not apply to the real life. For example, evidence is growing that thyroid autoimmunity, hypothyroidism or hyperthyroidism may manifest not only through the “classical” thyroid dysfunction syndrome, but also affecting postural equilibrium (1), mood (2), or handwriting (3). Nonetheless, recent research has pointed out that medications given for non-thyroidal illnesses (i.e., immune checkpoint inhibitors for cancer therapy) may have a great impact on the thyroid gland (4), and that coexistence of thyropathies with diseases of other organs may alter the clinical features of thyroid illnesses, the medications usually given to treat them, and their dosage (5). Finally, anatomical or morphological anomalies of the gland, uncommon cytological and histological features, or new gene mutations underlying neoplasm should contribute to the atypical presentation of thyroid disorders (6).

The scope of the present Research Topic—including 10 case reports, 4 review articles and 2 original research papers—was to provide new insights in the field of clinical-pathological manifestations of thyroid disorders.

Giuliani et al. reviewed the involvement of nuclear factor-kappa B (NF-kB)—an ubiquitous transcription factor involved in inflammatory and immune responses, and also in regulation of expression of many other genes related to cell survival, proliferation, and differentiation —in thyroid autoimmunity (included Graves' orbitopathy), thyroid cancer, and thyroid-specific gene regulation. Interestingly, this review has shown that, in thyroid cancer, the increased activity of NF-kB correlates with a more aggressive pattern.

Keeping to the topic of autoimmunity, Yao et al. investigated the expression of IL-36α mRNA in peripheral blood mononuclear cells from newly diagnosed patients with Graves' disease (GD), refractory GD patients and normal controls. They concluded that IL-36α and CD4+IL-36α+T cells may be involved in the pathogenesis of GD by promoting the production of Th1, Th2, and Th17 cytokines.

Hashimoto's thyroiditis (HT) and its relationship with thyroid cancer in children are reviewed by Esposito et al. Analyzing the literature, the authors state that children with HT should be considered at higher risk for thyroid cancer development and discuss the possible reasons of such coexistence.

Benvenga et al. report increased requirement of daily doses of L-thyroxine in two patients with the atrophic variant of Hashimoto's thyroiditis and liver cirrhosis. Because of better intestinal absorption, L-T4 oral liquid formulation was able to circumvent the increased need of L-T4 in these patients.

Viola et al. introduce the subject of the unusual behavior of some thyroid cancers. They report the case of a patient presenting with structural recurrence of papillary thyroid cancer—identified by increasing levels of anti-Thyroglobulin antibodies—after 10 years from excellent response to initial treatment (total thyroidectomy and radioiodine remnant ablation).

Marina et al. have thoroughly worked up a patient with a huge high grade epitheliod angiosarcoma of the thyroid gland, which is a rare, aggressive, mesenchymal tumor with vascular differentiation. The patient is still alive at 62 month follow-up, following total thyroidectomy, resection of central and left compartment neck lymph-nodes, and chemotherapy with epirubicin and ifosfamide.

Alharbi et al. describe an unusual parathyroid carcinoma arising from a completely intrathyroidal parathyroid gland. This case should alert the Endocrinologists who deal with patients affected by symptomatic hypercalcemia and no parathyroid gland detectable in the neck, on the possibility of atypical intrathyroidal parathyroid neoplasm.

Similarly, Asa and Mete report a mammary analog secretory carcinoma (MASC), an unusual tumor of salivary gland type, presenting as thyroid nodule and mimicking papillary thyroid carcinoma. The intrathyroidal location of MASC may be explained by the occasional finding of salivary gland tissue within the thyroid. Thus, this lesion should represent a pitfall in the cytological and histological work-up of thyroid nodules.

The peculiar issue of nodule location within the thyroid gland is the topic of the paper by Pontieri et al. Assessing literature data and guidelines to plan the extension of surgery in a patient with cytologically indeterminate thyroid nodule, the authors found several studies supporting that the isthmus malignant lesions were associated with a higher rate of multifocality, capsular invasion, extrathyroidal extension and central lymph node metastases.

Paragliola et al. report two cases of apparently sporadic medullary thyroid carcinoma (MTC) associated with the variant in exon 2 of RET (Rearranged during Transfection) gene. As the most frequent RET protooncogene variants are located in exons 10, 11, and 13 through 16 of the RET gene, it is crucial to check also the unusual RET mutations arising from the exon 2, in order to identify hereditary forms of MTC wrongly classified as “sporadic.”

In their thorough review, Baloch and LiVolsi explain in detail the pathologic pictures associated with clinical and/or biochemical hyperthyroidism, recalling even the rarest and unusual lesions causing thyrotoxicosis, i.e., struma ovarii, gestational trophoblastic disease, TRH- and TSH-secreting tumors, malignant neoplasms. They also focus on hyperthyroidism associated with antineoplastic agents and targeted therapies, which was the case of the two patients reported by Iadarola et al. The authors describe their experience with thyrotoxicosis induced by nivolumab, an immune checkpoint inhibitor. Thyroid dysfunction in both patients presented with a low serum level of TSH. However, endocrine evaluation showed a completely different etiology and clinical evolution.

In their research paper, Paragliola et al. evaluate the time to TSH normalization, on a specific L—T4 therapy dose regimen, in patients undergone total thyroidectomy for Graves' disease The authors have demonstrated that time to normalization of TSH may be prolonged, particularly in subjects with either longer duration of the disease before surgery, and high values of anti-TSH receptor autoantibodies (TrAb) at the diagnosis of hyperthyroidism.

Urselli et al. discuss in detail the risk to benefit ratio of treatment options in a patient affected by moderate-to-severe Graves orbitopathy with high clinical activity score, associated to uncontrolled type 2 diabetes mellitus. Based on a well-thought out choice, a regimen of low dose methylprednisolone administration plus fractionated low-dose orbital radiotherapy should be effective and better tolerated.

Finally, Sørensen et al. performed a systematic review on the impact of goiter and thyroidectomy on esophageal anatomy, esophageal physiology, and subjective swallowing dysfunction. They found that thyroidectomy relieved patients with goiter from dysphagia, within 6 months of surgery, probably via increase in the cross-sectional area of the esophagus.

Taken together, these studies have shown the multiform appearance of thyroid disorders, the complexity of clinical and therapeutic approach to thyroid patients, and the need of further research to bring to light thoroughly what hides under the tip of this intriguing iceberg.

## Author Contributions

All authors listed have made a substantial, direct and intellectual contribution to the work, and approved it for publication.

### Conflict of Interest Statement

The authors declare that the research was conducted in the absence of any commercial or financial relationships that could be construed as a potential conflict of interest.
